# Synergistic Removal of Cr(VI) Utilizing Oxalated-Modified Zero-Valent Iron: Enhanced Electron Selectivity and Dynamic Fe(II) Regeneration

**DOI:** 10.3390/nano15090669

**Published:** 2025-04-28

**Authors:** Song Hou, Jiangkun Du, Haibo Ling, Sen Quan, Jianguo Bao, Chuan Yi

**Affiliations:** 1 Hubei Provincial Academy of Eco-Environmental Sciences, Wuhan 430072, China; housong@hbaes.ac.cn (S.H.); linghaibo@hbaes.ac.cn (H.L.); quansen@hbaes.ac.cn (S.Q.); 2 Hubei Key Laboratory of Pollution Damage Assessment and Environmental Health Risk Prevention and Control, Wuhan 430072, China; 3 School of Environmental Studies, China University of Geosciences, Wuhan 430074, China; bjianguo@cug.edu.cn

**Keywords:** nano zero-valent iron, sodium oxalate, Cr(VI), reduction, electron transfer

## Abstract

To address the challenges of environmental adaptability and passivation in nanoscale zero-valent iron (nFe^0^) systems, we developed oxalate-modified nFe^0^ (nFe_oxa_) through a coordination-driven synthesis strategy, aiming to achieve high-efficiency Cr(VI) removal with improved stability and reusability. Structural characterization (STEM and FT-IR) confirmed the formation of a FeC_2_O_4_/nFe^0^ heterostructure, where oxalate coordinated with Fe(II) to construct a semiconductor interface that effectively inhibits anoxic passivation while enabling continuous electron supply, achieving 100% Cr(VI) removal efficiency within 20 min at an optimal oxalate/Fe molar ratio of 1/29. Mechanistic studies revealed that the oxalate ligand accelerates electron transfer from the Fe^0^ core to the surface via the FeC_2_O_4_-mediated pathway, as evidenced by EIS and LSV test analyses. This process dynamically regenerates surface Fe(II) active sites rather than relying on static-free Fe(II) adsorption. XPS and STEM further demonstrated that Cr(VI) was reduced to Cr(III) and uniformly co-precipitated with Fe(II/III)-oxalate complexes, effectively immobilizing chromium. The synergy between the protective semiconductor layer and the ligand-enhanced electron transfer endows nFe_oxa_ with superior reactivity. This work provides a ligand-engineering strategy to design robust nFe^0^-based materials for sustainable remediation of metal oxyanion-contaminated water.

## 1. Introduction

Chromate (Cr(VI)) contamination in water has long been seen as a critical issue globally. Chromium primarily exists in the form of Cr(III) and Cr(VI). Cr(III) has low toxicity and is recognized as a crucial trace element for mammals, while Cr(VI) possesses approximately 1000 times greater toxicity than the Cr(III) species [1]. Improper intake or long-term exposure of Cr(VI) can cause respiratory cancer, reproductive difficulties, and severe liver damage; thus, Cr(VI) has been recognized as a dangerous pollutant with the highest priority [2,3]. Therefore, efficient treatment of Cr(VI)-bearing wastewater has become as a vital task for sustainable industrial activities in the fields of electroplating, ore mining, metalworking, and leather manufacturing [4,5,6].

In the most recent decades, nanoscale zero-valent iron (nFe^0^) has been demonstrated to be an effective and eco-friendly material for in situ chemical remediation of Cr(VI)-contaminated sites owing to its reductive capability [7,8]. However, the advantages of nFe^0^ are also accompanied by characteristics of unstable and easy aggregation, owing to strong surface energy of nanoparticles, the weaker van der Waals force action, and the magnetic properties of iron [9,10,11]. A typical phenomenon of this is that nFe^0^ is highly susceptible to air passivation with formation of a surface oxide film, which can hinder the electron transfer and substance diffusion in the solid–liquid interface, resulting in decreased adsorption capacity and reductive reactivity of nFe^0^ [12,13,14]. Consequently, it is of practical importance to improve the inter-particle dispersion and stability of nFe^0^ particles.

To enhance the reactivity against surface passivation, numerous studies have focused on the modification of nFe^0^ at the level of material surface or the entire body to augment interface electronic transfer [15,16]. There may be three ways for electrons to transfer from the iron core to the surface, where they react with heavy metal ions: (1) direct electron transfer through surface defects of nFe^0^ particles, (2) a semiconductor composed of Fe_2_O_3_ and FeOOH iron oxide for electron transfer, (3) Fe(II) on the surface of nFe^0^ serves as an electron donor [17]. Normally, the outer iron oxide shell of nFe^0^ is covered by hydrophilic hydroxyl groups due to the spontaneous hydrolysis of nFe^0^ [18], leading to a low electron utilization efficiency of nFe^0^.

To date, a variety of approaches have been intensively investigated to modify nFe^0^ for enhanced pollutant degradation and electron selectivity, such as bimetallic iron, surfactant modification, and sulfidation [19,20,21]. For instance, the formation of bimetallic nanomaterials (M^0^/Fe^0^) can avoid the wasteful oxidation of nFe^0^ with water [22,23], increase the catalytic production of reactive atomic hydrogen, and decrease the deposition of corrosion byproducts, thereby accelerating the reduction kinetics towards target pollutants [24]. Furthermore, attempts of surface surfactant coating, such as polyelectrolytes and biopolymers [25,26], can effectively control the nucleation and aggregation of nFe^0^ particles, therefore, increasing reactivity by enhancing the iron-contaminant interaction [27]. Arunkumar et al. [28] have verified that the modification with polyvinyl pyrrolidone (PVP) and carboxymethyl cellulose (CMC) can improve the steric hindrance, stability, dispersion and mobility of nFe^0^ particles. Moreover, the sulfidation of nFe^0^ using different vulcanizing agents (Na_2_S, Na_2_S_2_O_3_, and Na_2_S_2_O_4_) has attracted particular interest in recent years. It was suggested that thiol groups would partly replace surface hydroxyl groups during nFe^0^ sulfidation in order to improve the electron selectivity and electron utilization efficiency of nFe^0^ for the removal of contaminants [29]. However, these existing modification methods also have some drawbacks in terms of cost, effectiveness and clean production. For instance, bimetallic nanoparticles are too expensive for large-scale application, while surfactant or sulfidated modification may result in secondary pollution of organics and H_2_S, respectively, as well as high selectivity toward some specific pollutants [30,31,32]. On this account, the development of alternative nFe^0^-based materials with an environmentally benign nature is highly desired and of great interest to actuate wider applications.

In addition to the aforementioned modifiers, low-molecular-weight organic acids (LMWOAs), including lactic, citric, malic and oxalic acids, have drawn particular attention in material modification in recent years. Typically, LMWOAs are widely available in natural environments [33,34]. They can be produced from plant root secretions, soil organic matter decomposition, and microbial metabolites, and are typically characterized by one or more carboxyl groups, with strong chelating capacity with multivalent metal cations [35,36]. Therefore, LMWOAs are expected to alter the surface charge of nFe^0^, thus reducing the tendency of particle agglomeration, or alleviating iron passivation through the formation of complex compounds [37,38]. Yuan et al. [39] demonstrated that S-nFe^0^ and LMWOAs exhibited significant synergistic promotion in the removal of Cr(VI). Further studies showed that LMWOAs promoted various aspects of diffusion, adsorption and complexation of Cr(VI) on the S-nFe^0^ surface, which, in turn, improves the electron selectivity for Cr(VI). Furthermore, tartrate or malate were reported to enhance the adsorption of Cr(VI) in the presence of S-nFe^0^ and accelerate the dissolution of the iron passivation layer through the formation of reactive nFe^0^-ligand systems either under anoxic or aerobic conditions [40,41]. In addition, compared to other LMWOAs, OA is non-toxic and harmless and can promote the dissolution of Fe(II) under acidic conditions, while C_2_O_4_^2−^ has a π-conjugated structure, and the delocalized electrons of C_2_O_4_^2−^ are favorable for the reduction of Fe(III) to Fe(II) [42,43,44]. Meanwhile, as a clean and effective remediation agent, oxalic acid is effective in improving pollutant degradation and removal efficiency. Wu et al. [45] found that the oxidized layer on the Fe^0^ surface was replaced by a ferrous oxalate shell layer through the ball milling process, accelerating the dissolution of Fe^0^ to generate Fe(II) for PS activation, which can significantly degrade pollutants. Li et al. [46] reported that the removal efficiency of Cr(Ⅵ) from wastewater can be effectively improved by using waste biomass N-doped lignocellulosic charcoal as a catalyst and OA as media. In addition, OA could also facilitate the corrosion of nFe^0^, accelerating the release of Fe(II). Since the redox potential of Cr(VI)/Cr(III) (1.33 V vs. NHE) is higher than that of Fe(III)/Fe(II) (0.77 V vs. NHE), a large amount of Fe(II) is produced, which significantly enhances the heterogeneous and homogeneous reduction of Cr(VI) in solution [39]. Therefore, the electron transfer pathway and the electron selectivity of Fe^0^ are also changed accordingly during the removal of Cr using OA-modified promoted Fe^0^. However, studies on the electron transfer capacity of oxalate-modified nFe^0^ have been very limited to date. Whether the primary pathway of electron transfer for Cr(VI) reduction by nFe^0^ is directly from nFe^0^ to Cr(VI) or from Fe(II) to Cr(VI) after the dissolution of nFe^0^, the process is currently unknown, which would be an obstacle to the application of nFe_oxa_ on the remediation of Cr(VI).

In order to address this gap, the objective of this research was to enhance the Cr(VI) removal property of nFe^0^, and the performance of nFe_oxa_ at different C_2_O_4_^2−^/Fe molar ratios was tested from the perspective of reductive Cr(VI) removal. This study examined how different positive anions, oxygenated anion, humic acid, and the lifespan of nFe_oxa_ influence its ability to adapt to the aquatic ecosystem. Finally, the reaction mechanism in regard to the synergistic removal of Cr(VI) was unveiled using various tools, including fundamental electrochemical analysis and solid characterizations. This study presented a new insight into Cr(VI) removal, which was beneficial to understand the application boundaries of nFe_oxa_ for Cr(VI) remediation, and provided more theoretical guidance for the subsequent oxalic acid-modified materials.

## 2. Materials and Methods

### 2.1. Chemicals and Materials

Sodium borohydride (NaBH_4_), sodium sulfide (Na_2_S·9H_2_O), iron sulfate (FeSO_4_·7H_2_O), sodium oxalate (Na_2_C_2_O_4_), potassium bichromate (K_2_Cr_2_O_7_), sulfuric acid (H_2_SO_4_), sodium hydroxide (NaOH), diphenyl carbamide (C_13_H_14_N_4_O), humic acid (HA), sodium carbonate (Na_2_CO_3_), and ethanol were purchased from Sinopharm Chemical Reagent Co., Ltd. (Shanghai, China) at an analytical grade. The deoxygenated water was prepared by purging deionized water with pure nitrogen for 1 h prior to use.

### 2.2. Preparation of Oxalated nFe^0^

The oxalated nano-scale zero-valent iron particles (nFe_oxa_) were prepared through the liquid phase reduction method. Briefly, ferrous sulfate, sodium oxalate and NaBH_4_ were dissolved in deoxygenated water, respectively. Then, the NaBH_4_ solution was mixed with the solution of sodium oxalate to obtain the NaBH_4_/Na_2_C_2_O_4_ mixture, which was added dropwise into the FeSO_4_ solution in a three-necked flask equipped with mechanical stirring under anaerobic conditions. After stirring for 20 min, FeSO_4_ was fully reduced to zero-valent iron. The black particles generated in the three-neck flask were then isolated using a magnet, thoroughly washed three times with distilled water and ethanol, and ultimately dried in a vacuum oven at 60 °C for 12 h. As expected, nFe_oxa_ particles with different C_2_O_4_^2−^/Fe^2+^ molar ratios, namely 1/14, 1/19, 1/29 and 1/58, were obtained and flagged as nFe_oxa_(x/y), where x and y refer to the molarity of C_2_O_4_^2−^ and Fe^2+^, respectively. For instance, nFe_oxa_(1/29) denotes oxalated nFe(0) with a C_2_O_4_^2−^/Fe^2+^ molar ratio of 1/29.

### 2.3. Batch Experiments of Cr(VI) Removal

The batch experiments of Cr(VI) removal were carried out in a 250 mL beaker containing 100 mL of the 10 mg/L Cr(VI) solution. The initial solution pH was adjusted with diluted HCl and NaOH. To initiate the reaction, 0.1 g of nFe_oxa_ particles was injected into the beaker, and the reaction solution was thoroughly mixed by mechanical stirring (300 rpm) at ambient conditions. At specified time intervals, a 1 mL sample was taken and subsequently filtered using a 0.22 μm polyethersulfone filter before analysis.

This study examined how the C_2_O_4_^2−^/Fe^2+^ molar ratio (1/14, 1/19, 1/29 and 1/58), the dosage of the material (ranging from 0.3 to 1.5 g/L), the presence of co-existing anions and humic acid (ranging from 10 to 80 mg/L), as well as the initial concentration of Cr(VI) (10–50 mg/L) affected the removal of Cr(VI). In addition, the stability property of nFe_oxa_ after the reaction was further evaluated. Typically, the used nFe_oxa_ particles were separated from the reacted solutions with the help of the magnet and washed with deoxygenated DI water, and then the fresh Cr(VI) solution was added to react with the used Fe_oxa_ particles once again.

### 2.4. Analysis

Characterization of the nFe_oxa_ particles, both prior to and following their reaction with Cr(VI), was performed using Transmission Electron Microscopy (TEM, CM12/STEM, Philips, Amsterdam, The Netherlands) equipped with an energy-dispersive spectrometer (EDS, Themis Z, Thermo Fisher Scientific, Waltham, MA, USA), X-ray Diffraction (XRD, X’Pert Pro, PANalytical, Malvern, UK), Scanning Transmission Electron Microscopy (STEM, Quanta 450, Thermo Fisher Scientific, Waltham, MA, USA) with high-angle annular dark-field imaging and phase-mapping capabilities, Brunauer–Emmett–Teller (BET) surface analysis (ASAP 2020, Micromeritics, Norcross, GA, USA), Fourier-Transform Infrared Spectroscopy (FT-IR, Nicolet iS50 ABX, Thermo Fisher Scientific, Waltham, MA, USA), X-ray Photoelectron Spectroscopy (XPS, VG Multilab 2000, VG Scientific, Edgewood, MD, USA) and various electrochemical tests. A three-electrode system was utilized for electrochemical analyses, operating within a voltage range of −2.0 V to 2.0 V at a scanning of 0.05 V s^−1^ employing a VersaSTAT 3. The working electrode consisted of a stainless-steel mesh coated with particles (1 cm^2^), while Ag/AgCl and Pt wire served as the reference and counter electrodes, respectively. For all electrochemical experiments, 0.1 M Na_2_SO_4_ acted as the electrolyte. The concentrations of Cr(VI) in the aqueous solution were quantified through the 1,5-diphenylcarbazide colorimetric method, measured at 540 nm using a UV–visible spectrophotometer (UV/Vis, UV5500PC, Shanghai Yuanxi Instrument Co., Ltd., Shanghai, China), demonstrating a detection limit of 0.004 mg/L and a linear range up to 1.0 mg/L. Additionally, the aqueous concentration of Fe(II) was determined via the 1.10-phenanthroline colorimetric method at a wavelength of 510 nm on the same spectrophotometer.

## 3. Results and Discussion

### 3.1. Characterization of nFe_oxa_

**Figure 1:**
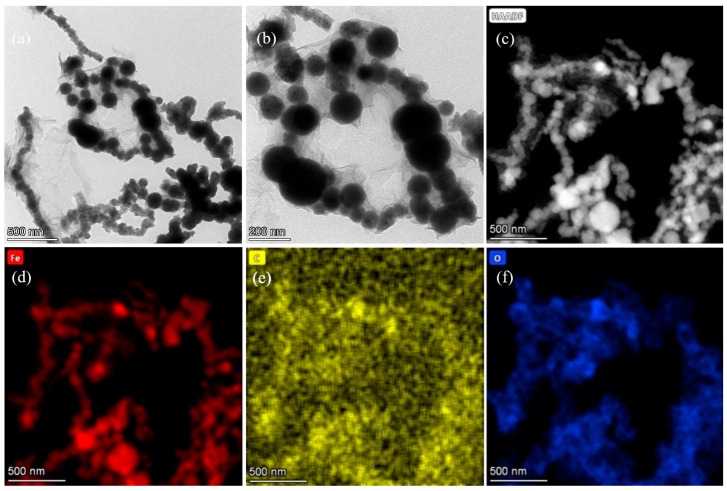
(**a**,**b**) TEM analysis of fresh nFe_oxa_ (S/Fe molar ratio = 1/29), (**c**) STEM image, (**c**–**f**) HAADF-STEM mappings of fresh nFe_oxa_(1/29) (elements Fe, C, and O are denoted in red, yellow, and blue).

The pristine nFe_oxa_’s morphology and microstructure were analyzed using TEM and STEM imaging (Figure 1a,b), along with phase mapping. The fresh nFe_oxa_ exhibited a characteristic chain structure [47]. From the TEM and STEM images, typical chain-like formations were retained in the fresh nFe_oxa_, while spherical and rectangular shapes were observed due to aggregation and compression. When nFe^0^ was modified with oxalic acid, nFe_oxa_ was surrounded by a muslin-like lamellar structure, which would be the oxalate–iron complexes.

The STEM element map of nFe_oxa_ (Figure 1c,f) indicates that Fe species highly overlapped with the O element, whereas the C element is highly dispersed. Noteworthily, the signal of the C element enhanced in sites overlapped with the Fe element, indicating the coordination and complexation of oxalic acid with Fe(II). This is similar to the distribution law of the “tissue” observed in the TEM image, and it illustrates the success of the doping of sodium oxalate and the formation of oxalate–iron complexes.

**Figure 2:**
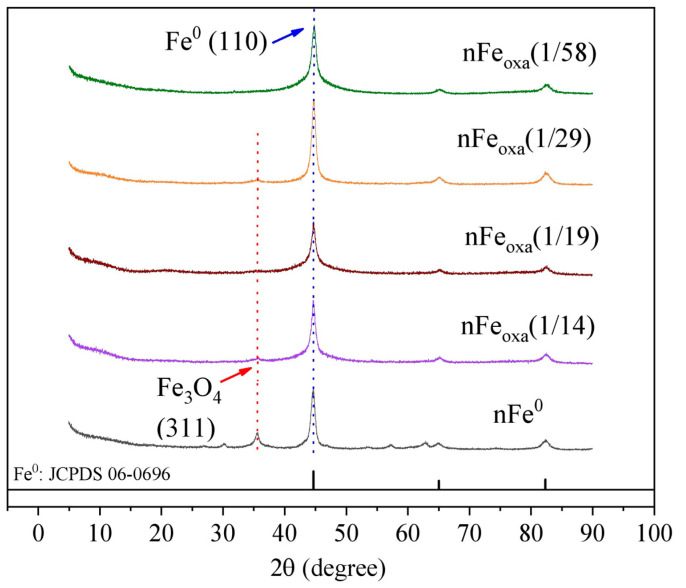
X-ray diffraction patterns of nFe^0^ and nFe_oxa_ particles.

To further delve into the effect of oxalic acid doping in nFe_oxa_, the mineral composition and crystallinity of nFe^0^ and nFe_oxa_ were compared by XRD characterization. As depicted in Figure 2, nFe^0^ both before and after oxalic acid modification exhibits a typical zero-valent iron diffraction peak at 44.6°. Weak characteristic diffraction peaks of Fe_3_O_4_ can be seen in the XRD spectrum of bare nFe^0^, signifying the presence of a thin Fe_3_O_4_ oxide layer on the surface of nFe^0^. However, there is no corresponding magnetite diffraction peak in the oxalic-acid-modified nFe_oxa_ material, indicating that the doping of oxalic acid can avoid the oxide layer formation on the surface of nFe^0^, thereby postponing the passivation process. Moreover, in conjunction with the outcomes of TEM and STEM, the passivation process was further constrained simultaneously by the existence of the oxalate–iron complexes. Here, the doping of oxalate acid can not only preserve the Fe(II) active sites of nFe_oxa_, but also increase the potential for the contact of pollutants with nFe_oxa_.

**Figure 3:**
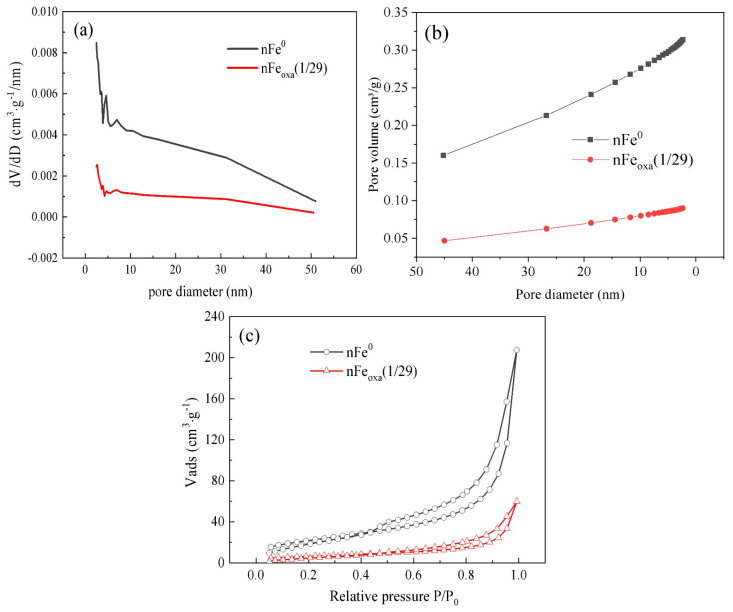
(**a**) Particle pore size distribution map; (**b**) cumulative pore volume map; (**c**) N_2_ adsorption and desorption isotherms of nFe^0^ and nFe_oxa_.

The N_2_ sorption and desorption properties of nFe^0^ before and after oxalation were characterized, both of which showed type IV isotherm with an H3 hysteresis loop, as depicted in Figure 3, indicating a typical monolayer adsorption. Regardless of oxalation or not, most of the pores of nFe^0^ had diameters of 0–10 nm, ascribed to mesoporous particles. The BET surface areas of the nFe_oxa_(1/29) and nFe^0^ materials were determined to be 23.52 m^2^/g and 80.63 m^2^/g, respectively, while their material pore volumes were 0.090041 cm^3^/g and 0.313722 cm^3^/g (Table 1). These results indicated that bare nFe^0^ without oxalate modification possessed a significantly larger specific surface area and pore volume than its oxalated counterpart. In contrast to the assumption that a smaller specific surface area would be commonly accompanied by less chemical adsorption and reductive sites on the material surface would also be reduced, the reactivity of nFe_oxa_ herein was not attributed to a larger surface area but caused by a potential synergistic effect within the composite.

**Figure 4:**
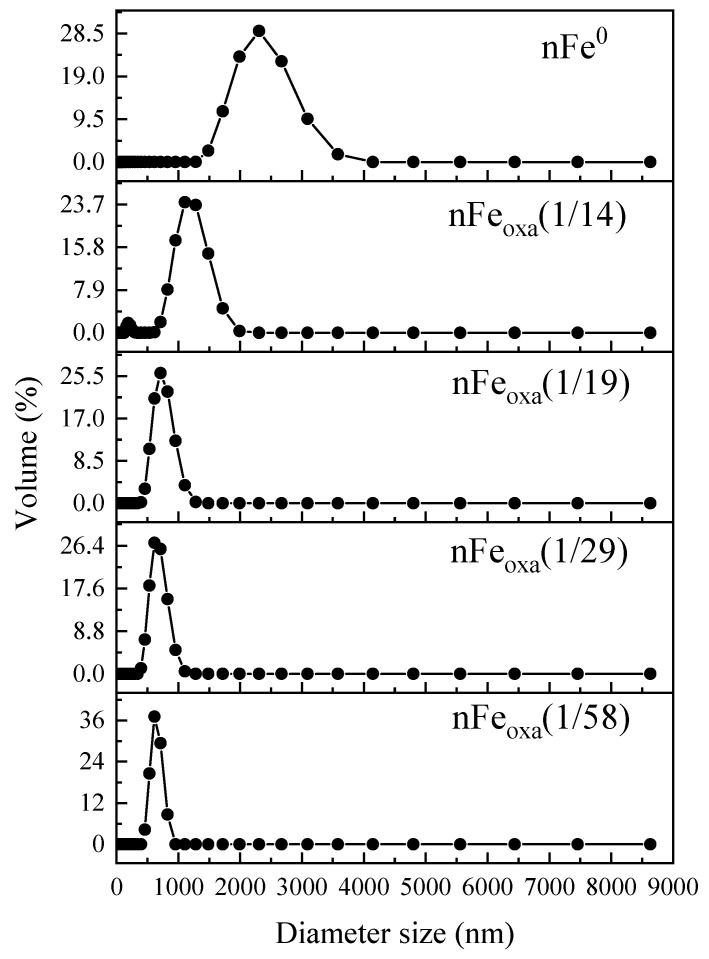
Particle size distribution of nFe^0^ and nFe_oxa_.

By comparing the particle size of the nFe_oxa_ and nFe^0^ materials (Figure 4), the particle size of unmodified nFe^0^ particles is primarily concentrated around 3000 nm, while the particle sizes of nFe_oxa_ modified with oxalic acid become smaller and decrease with the reduction in the oxalic C_2_O_4_^2−^/Fe^2+^ molar ratio. As presented in Table 2, the average particle size of nFe^0^ in this experiment is about 4–5 times that of the average particle size of the modified nFe_oxa_ composites. The smaller particle size is beneficial to the migration and diffusion in water, and the stronger Brownian motion is beneficial for contact with free Cr(VI) and to expedite the removal of Cr(VI). From the characterization results above, it can be observed that oxalic acid doping has a significant impact on the morphology, crystal form, specific surface area and particle size of nFe^0^, leading to a discrepancy between the physicochemical properties and reactivity of nFe_oxa_ and nFe^0^. Consequently, it was essential to further elucidate the occurrence form of oxalic acid in nFe^0^.

**Figure 5:**
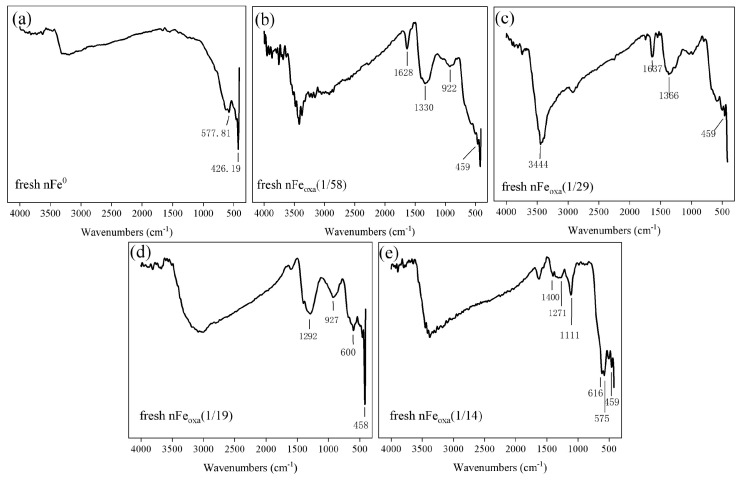
FT-IR spectra of (**a**) nFe^0^, (**b**) nFe_oxa_ (1/58), (**c**) nFe_oxa_ (1/29), (**d**) nFe_oxa_ (1/19), and (**e**) nFe_oxa_ (1/14) of fresh nFe^0^ and nFe_oxa_ materials.

The FT-IR results of nFe_oxa_ and Fe^0^ are presented in Figure 5. Compared to single nFe^0^, the newly emerged FT-IR spectra of 1620–1640 cm^−1^, 1270–1360 cm^−1^ and 1111 cm^−1^ can be recognized as consistent with the FT-IR vibration peaks of iron–oxalic acid complexes as reported previously [48]. The peak at wavenumber of 577 cm^−1^ and 927 cm^−1^ suggests the existence of magnetite and goethite on the material surface. This is consistent with the results of STEM and XRD, demonstrating the appearance of FeC_2_O_4_ and its coexistence with Fe(0) during nFe_oxa_ formation.

### 3.2. Effect of Different S/Fe Molar Ratios

**Figure 6:**
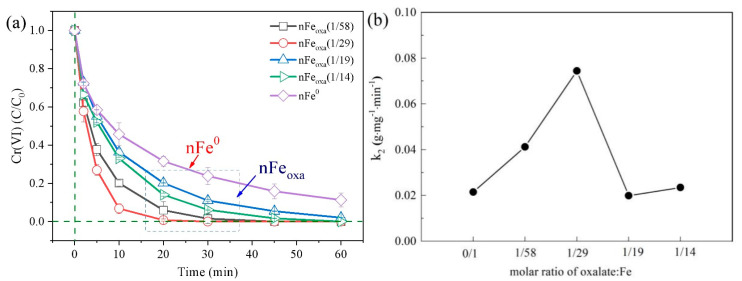
(**a**) Cr(VI) removal effect of nFe_oxa_ with different doping ratios of oxalic acid; (**b**) second-order kinetic constant change diagram.

As illustrated in Figure 6a, the nFe_oxa_ composite with varying Fe^2+^/C_2_O_4_^2−^ molar ratios demonstrated significantly higher Cr(VI) removal rates compared to pristine nFe^0^. Due to strong coordination and chelation ability [43], C_2_O_4_^2−^ possessing two carboxyl groups could coordinate with iron atoms on the surface of iron oxides to form diverse iron oxide composite products [49,50,51]. Additionally, C_2_O_4_^2−^ with conjugated π bond would be advantageous for the surface Fe(III) to obtain electrons from the iron core and then convert to Fe(II) [52]. The rapid electron transfer among Fe(0), Fe(III) and Fe(II) in the reaction interface is beneficial for the adsorption and reduction of Cr(VI), as well as the co-precipitation of Cr(III) with Fe(III) on the particle surface. When the molar ratio of C_2_O_4_^2−^/Fe^2+^ was 1/29, the prepared nFe_oxa_(1/29) achieved the highest reactivity and completely removed Cr(VI) within 20 min. However, under the same conditions, the removal efficiency of Cr(VI) by nFe^0^ was only 68.5% in 20 min, and 88.7% after 1 h reaction. Furthermore, the results of the pseudo-second-order kinetic constants (Figure 6b) showed that the reactivity of nFe_oxa_ was not linearly related to the doping amount of oxalic acid. The removal rate of Cr(VI) initially increased and then decreased with the increase in oxalic acid, and ultimately remained stable [53,54]. This result may be due to the fact that C_2_O_4_^2−^ was negatively charged and showed a charge repulsion effect with Cr(VI). The oxalic acid was more likely to enhance the reduction activity and electron transfer rate of nFe^0^, and the change brought about by the oxalic acid doping also exists in an optimal balance. Therefore, in this study of the surface modification of micron zero-valent iron with oxalic acid, C_2_O_4_^2−^ dissolves in the liquid phase [48]. When the doping ratio of oxalic acid is too high, a large amount of free oxalate ions in the reaction solution may cause the nanoparticles to dissociate, resulting in a decrease in the adsorption capacity of Cr(VI).

### 3.3. Effect of Co-Existing Ions and Humic Acid

To investigate the impacts of co-existing water anions (such as PO_4_^3−^, CO_3_^2−^, NO_3_^−^, and Cl^−^) and humic acid (HA) on Cr(VI) removal by nFe_oxa_, both high and low concentrations were studied. According to previous reports, the co-existing ions in water can form surface metal complexes with the iron oxide layer on the surface of nFe^0^, which would promote the dissolution of the shell and enhance electron transfer [55,56,57]. Specifically, PO_4_^3−^ and CO_3_^2−^ can chelate, precipitate and passivate the ferrous irons, while NO_3_^−^ can be reduced by nFe^0^ [58]. Cl^−^ can promote the corrosion process of zero-valent iron, thereby accelerating the passivation of zero-valent iron [59,60].

**Figure 7:**
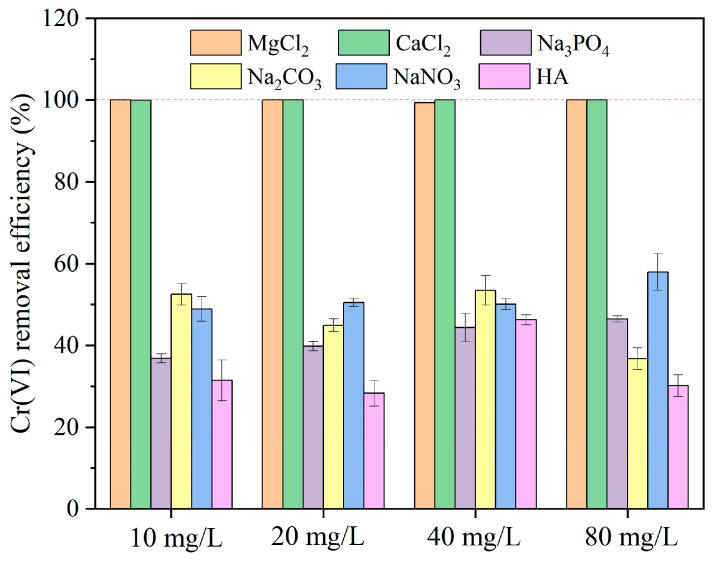
Effect of water anions and humic acid on Cr(VI) removal by nFe_oxa_ (reaction conditions: [nFe_oxa_] = 1.0 g/L (C_2_O_4_^2−^/Fe^2+^ molar ratio = 1/29), [Cr(VI)] =10 mg/L, initial pH = 6, T = 25 °C).

As illustrated in Figure 7, Ca^2+^ and Mg^2+^ showed an insignificant effect on the removal of Cr(VI), where Cr(VI) can be completely removed. Previous studies demonstrated that the removal of pollutants in the system by metal cations is affected by the radius of their hydrated ions [61]. The radius sizes of Ca^2+^, Mg^2+^, and Cr^6+^ are 99 pm, 72 pm and 52 pm, respectively. Accordingly, a smaller ionic radius and higher charge would correspond to a greater degree of ion polarization and adsorption affinity [62]. At the same time, the addition of CaCl_2_ and MgCl_2_ introduces a significant amount of Cl^−^, thus promoting nFe_oxa_ corrosion and enhancing aqueous conductivity, as well as expediting the electron transfer between nFe_oxa_ and Cr(VI) to facilitate the Cr(VI) removal [59].

Nevertheless, PO_4_^3−^, CO_3_^2−^ and NO_3_^−^ all restrained the removal of Cr(VI) in the system. PO_4_^3−^ and CO_3_^2−^ were expected to compete with Cr(VI) for active sites on the surface of nFe_oxa_ and simultaneously form complexes and co-precipitates with iron oxides on the particle surface. For instance, PO_4_^3−^ can form Fe-PO_4_^3−^ minerals with Fe(II), while CO_3_^2−^ would be adsorbed onto the surface of nanoparticles, co-precipitate with Fe(II) as FeCO_3_, and occupy and deplete Fe(II) active sites, resulting in a reduction in the removal efficiency of Cr(VI) [63,64,65].

Additionally, NO_3_^−^ inhibited the reduction of Cr(VI) by competing for electrons. Since the standard electrode potential of NO_3_^−^/NO_2_^−^(0.01 V) is higher than that of Fe^2+^/Fe(−0.44 V), NO_3_^−^ can compete with CrO_4_^2−^ for Fe(II) and Fe(0) to be reduced to NO_2_^−^ [66,67,68]. Moreover, humic acid showed a remarkable inhibition to Cr(VI) removal by nFe_oxa_ (Figure 7). As viscous macromolecular organic matters in solution, humic acid can form complexes with surface iron species via abundant carboxyl functional groups, thus occupying nFe_oxa_ surface sites and impeding the interaction with Cr(VI) [69].

### 3.4. Reusability

**Figure 8:**
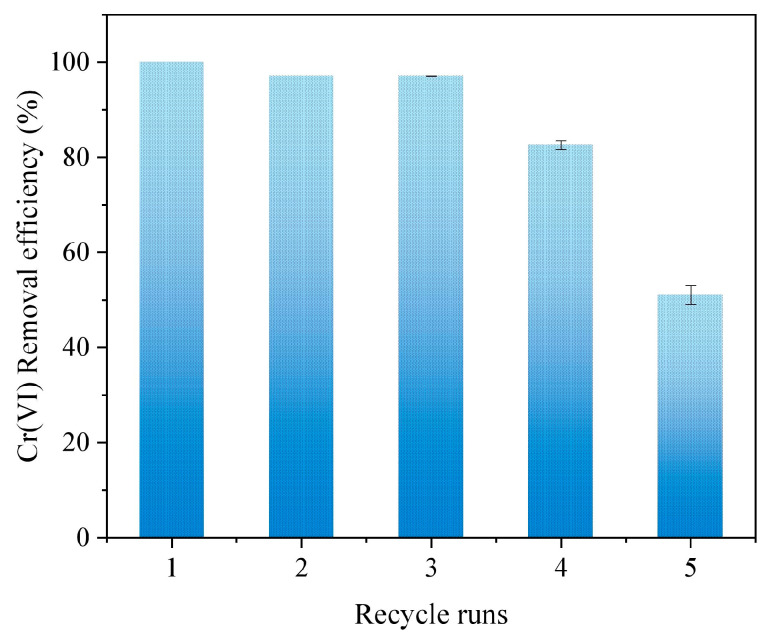
Reuse effect of nFe_oxa_ material in the Cr(VI) removal process.

Figure 8 presents the effect of five reuse times on Cr(VI) removal by nFe_oxa_. The removal efficiency of Cr(VI) by nFe_oxa_ gradually decreased along the reuse times, which was nearly 50% after five times of reuse. According to previous reports, the removal rate of Cr(VI) dropped to 33.4% for the FeS-modified nFe^0^ material after three cycles, while the removal efficiency of Cr(VI) for nFe^0^ alone was 19.8% after three reuse runs. This finding indicated that nFe_oxa_ possessed good reusability in removing Cr(VI) [70].

**Figure 9:**
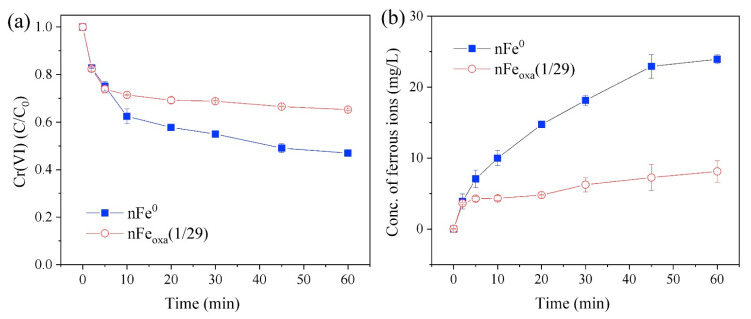
(**a**) The effect of 1.10-phenanthroline on the removal of Cr(VI) by nFe_oxa_ and nFe^0^, (**b**) release of Fe(II) with nFe_oxa_ and nFe^0^ of different S/Fe molar ratios in the presence of 1.10-phenanthroline.

Quenching Fe(II), recognized as a primary reductant and precipitator for Cr(VI), significantly influenced the sequestration of Cr(VI). As indicated in Figure 9a, the removal efficiencies for both nFe^0^ and nFe_oxa_ saw a considerable reduction compared to the reaction that excluded 1,10-phenanthroline. After adding 1,10-phenanthroline for 60 min, the removal efficiency of Cr(VI) by nFe^0^ and nFe_oxa_ decreased by approximately 40% and 70%, respectively. It was obvious that Fe(II) played an essential role in the removal of Cr(VI) from the aqueous solution, and the contribution of Fe(II) to the removal of Cr(VI) was higher in nFe_oxa_ than in nFe^0^. In other words, the nFe_oxa_ surface produced significantly more Fe(II) active sites than nFe^0^ during the reaction. Further analyzing the change in Fe(II) concentration in Figure 9b, under the same reaction conditions with 1,10-phenanthroline present, the behavior of aqueous Fe(II) diverged. The Fe(II) concentration of nFe^0^ continued to increase with the reaction, while the Fe(II) concentration of nFe_oxa_ increased slowly and then remained stable after adding 1,10-phenanthroline. After 60 min of reaction, the concentration of Fe(II) in nFe^0^ was significantly higher than that in nFe_oxa,_ indicating that nFe^0^ could produce more free Fe(II) for Cr(VI) reduction. However, nFe_oxa_ was based on the Fe(II) reactive component on the material surface for Cr(VI) reduction.

**Figure 10:**
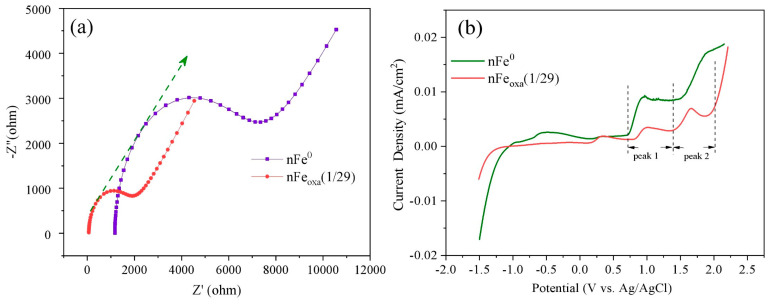
(**a**) EIS of nFe_oxa_ and nFe^0^, (**b**) CV of nFe_oxa_ and nFe^0^.

Based on previous results of the BET test, it is indicated that the specific surface area of nFe_oxa_ was smaller than that of nFe^0^. Thus, the enhanced reactivity of nFe_oxa_, relative to nFe^0^, is unlikely to be ascribed to an increase in surface active sites but may stem from a more rapid rate of electron transfer during the reaction. To explore the charge transfer characteristics of nFe0 and nFe_oxa_, electrochemical impedance studies (EISs) were conducted. As shown in Figure 10a, the larger curve indicated that the electrode exhibited lower charge transfer capability and higher resistance, while nFe_oxa_ generated a much smaller curve than nFe^0^, suggesting that the electron transfer resistance of nFe_oxa_ was lower than that of bare nFe^0^. The reason for this phenomenon may be that the formation of an iron oxide passivation layer is avoided during the H_2_C_2_O_4_ modification process, and the newly generated FeC_2_O_4_/Fe^0^ semiconductor structure is beneficial for the transfer of charges.

Furthermore, through the linear sweep voltammetry (LSV) test of the material (Figure 10b), two oxidation peaks (0.75–1.43 V and 1.43–2.0 V) can be found in the LSV spectra of nFe_oxa_ and nFe^0^, which were ascribed to be the oxidation peak of Fe(0)→Fe(II) and Fe(II)→Fe(III). When the potential corresponding to the oxidation peak of the material was smaller, the reaction proceeded more easily. Correspondingly, the potential of nFe_oxa_ in the oxidation peak of Fe(0)→Fe(II) is similar to that of nFe^0^, while the potential of nFe_oxa_ in the oxidation peak of Fe(II)→Fe(III) is slightly smaller than that of nFe^0^, indicating that Fe(II) in nFe_oxa_ more easily acts as an electron donor to transfer electrons to Cr(VI).

### 3.5. Mechanism of Cr(VI) Sequestration by nFe_oxa_

**Figure 11:**
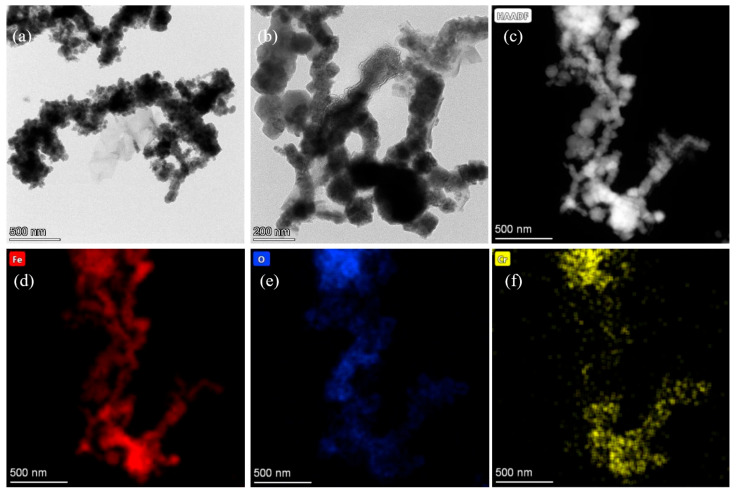
(**a**,**b**) The TEM results of Cr-treated nFe_oxa_(1/29), (**c**) STEM image, (**c**–**f**) HAADF-STEM mappings of Cr-treated nFeoxa(1/29) (elements Fe, O and Cr are denoted in red, blue and yellow).

The characteristics of nFe_oxa_ following reactions were analyzed through the TEM, STEM and XPS techniques to gain a clearer insight into the reaction mechanism. The TEM image (Figure 11a,b) indicated that the surface morphology of the Cr-treated nFe_oxa_ had changed considerably compared with that of the fresh nFe_oxa_. The fresh nFe_oxa_ material had an obvious spherical shape and smooth surface, while the Cr-treated nFe_oxa_ had a significant tendency to agglomerate, with the boundary gradually blurring and the surface passivation layer apparently thickening. Numerous smaller particles formed on the surface of nF_eoxa_, presumably caused by the formation of Fe-Cr strong oxides attached to the surface of the material after the reduction of Cr(VI). Meanwhile, in conjunction with the STEM elemental mapping of the reacted nFe_oxa_ material (Figure 11c–f), it can be clearly observed that the Cr element was highly overlapped with the Fe and O elements, indicating that Cr was enriched on the nFe_oxa_ surface.

**Figure 12:**
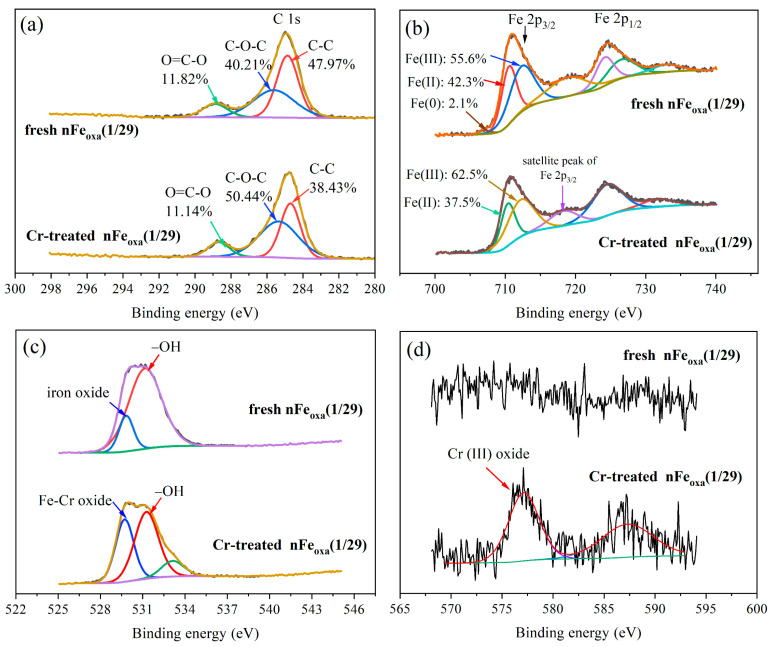
XPS spectra of (**a**) C 1s, (**b**) Fe 2p, (**c**) O 1s, and (**d**) Cr 2p of the fresh and Cr-treated nFe_oxa_.

Further investigation into the speciation of carbon (C), iron (Fe), oxygen (O), and chromium (Cr) in nFe_oxa_ prior to and post-reaction was conducted utilizing XPS. It can be seen from Figure 12a that the C 1s at 284.86 eV can be ascribed to the characteristic peak of the carboxyl group O=C-O in the molecular structure of oxalic acid. The C content in O=C-O before and after the reaction was 11.82% and 11.14%, respectively, indicating that its content remained basically stable, with only a very small amount of oxalate lost during the reaction. As presented in Figure 12b, Fe 2p corresponded to the Fe 2p_3/2_ characteristic peaks of Fe(0), Fe(II) and Fe(III) at the binding energies of 707.03 eV, 710.55 eV and 712.34 eV, respectively. After the reaction, the characteristic peak of Fe(0) disappeared, and the atomic specific gravity of Fe(II) decreased, while that of Fe(III) increased, indicating that Fe(0) and Fe(II) on the surface of the material were finally oxidized to Fe(III). As shown in Figure 12c, the strength of O-H bonds was significantly weakened, and the proportion of the metal-O bonds increased due to the rusting of nFe^0^ from iron oxides containing Fe-O bonds and Fe-Cr-O bonds. In addition, Figure 12d indicates that the peaks with binding energies of 577.0 eV and 586 eV in the Cr 3d spectrum corresponded to low toxicity Cr(III), thus confirming the conversion of Cr(VI) to Cr(III). From these extensive findings, it can be inferred that there exists a hierarchical material consisting of an Fe^0^ core, an FeC_2_O_4_ shell, and Fe or Fe/Cr-oxide precipitates.

**Figure 13:**
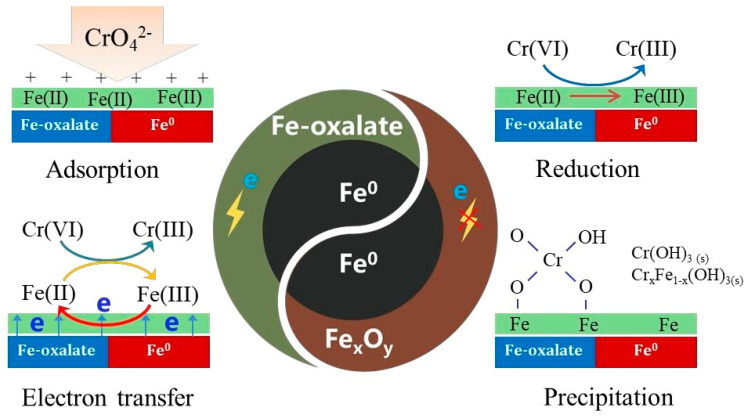
Mechanism for Cr(VI) removal by nFe_oxa_.

As stated, the mechanisms of removal process of Cr(VI) by nFe_oxa_ mainly involved adsorption, reduction, electron transfer, and co-precipitation, as shown in Figure 13. During the preparation of nFe_oxa_, oxalate would form a complex with Fe(II), and together with the nFe^0^ crystal nucleus, form a composite material of ferrous oxalate and zero-valent iron. This composite structure endowed nFe_oxa_ with higher stability than nFe^0^, and decreased the surface iron oxide passivation layer. According to the electrochemical characterization results, more charges in nFe_oxa_ could migrate from the Fe^0^ core to surface, which conformed to the theoretical speculation regarding the formation of “d-p” conjugation between the “p” orbital of the conjugated π bond in the oxalate and the “d” orbital of the Fe atom to enhance the electron cloud density of the iron atom. This enhancement in electron density also strengthened the adsorption of CrO_4_^2−^ by nFe_oxa_. Simultaneously, the surface Fe(II) active components were more likely to receive electrons migrated from Fe^0^ to be regenerated after the redox reaction with Cr(VI), instead of generating more surface free Fe(II) to achieve Cr(VI) restoration. Eventually the product of the redox reaction, Cr(III), was unstable in the aqueous solution and liable to form Cr(OH)_3_ and Fe-Cr hydroxide precipitates that would adhere and deposit on the surface of the material.

## 4. Conclusions

In the present study, oxalate-modified nano zero-valent iron (nFe_oxa_) was successfully synthesized through a liquid-phase reduction method, where oxalic acid acted as both a coordination ligand and a structural regulator to construct a FeC_2_O_4_/nFe^0^ heterostructure. The composite demonstrated exceptional Cr(VI) removal efficiency (100% within 20 min at an optimal oxalate/Fe molar ratio of 1/29), outperforming conventional nFe^0^ systems. Key findings include:

Electron transfer enhancement: Electrochemical analyses (EIS, LSV) revealed that oxalate ligands facilitated rapid electron transport from the Fe^0^ core to the surface, dynamically regenerating Fe(II) active sites. This mechanism compensated for the reduced specific surface area (BET data) and dominated the redox process.

Selective ion effects: divalent cations (Ca^2^⁺, Mg^2^⁺) promoted Cr(VI) reduction via electrostatic interactions, while oxyanions (e.g., PO_4_^3^⁻) and humic acid competitively inhibited reactivity by blocking surface sites.

Reusability and stability: nFe_oxa_ retained >50% Cr(VI) removal efficiency after 5 cycles—a significant improvement over previous systems (33.4% decline), underscoring its environmental compatibility.

Immobilization mechanism: STEM mapping and XPS spectra confirmed that Cr(VI) was reduced to Cr(III) and immobilized through co-precipitation with Fe(II/III)–oxalate complexes, effectively mitigating secondary contamination.

This work not only provides a ligand-engineering strategy to design durable nFe^0^-based materials but also decouples the roles of electron transfer kinetics and surface area in heavy metal remediation. The insights into semiconductor-mediated redox mechanisms offer a framework for optimizing nanomaterial design toward sustainable water treatment applications.

## Figures and Tables

**Table 1:** BET specific surface area and pore volume parameters of nFe^0^ and nFe_oxa_ particles.

Materials	BET Surface Area (m^2^/g)	Pore Diameter (nm)	Pore Volume (cm^3^/g)
nFe^0^	80.6138	15.9322	0.313722
nFe_oxa_(1/29)	23.5177	15.7567	0.090041

**Table 2:** Average particle size of nFe^0^ and nFe_oxa_ particles.

Materials	Particle Size D(50) (nm)
nFe^0^	2190
nFe_oxa_(1/14)	641
nFe_oxa_(1/19)	478
nFe_oxa_(1/29)	483
nFe_oxa_(1/58)	456

## Data Availability

All raw data are available upon request from the corresponding author.
